# Protozoan-Viral-Bacterial Co-Infections Alter Galectin Levels and Associated Immunity Mediators in the Female Genital Tract

**DOI:** 10.3389/fcimb.2021.649940

**Published:** 2021-08-05

**Authors:** Raina N. Fichorova, Allison K. DeLong, Susan Cu-Uvin, Caroline C. King, Denise J. Jamieson, Robert S. Klein, Jack D. Sobel, David Vlahov, Hidemi S. Yamamoto, Kenneth H. Mayer

**Affiliations:** ^1^Department of Obstetrics, Gynecology and Reproductive Biology, Harvard Medical School, Brigham and Women’s Hospital, Boston, MA, United States; ^2^Center for Statistical Sciences, School of Public Health, Brown University, Providence, RI, United States; ^3^Department of Obstetrics and Gynecology, Brown University, The Miriam Hospital, Providence, RI, United States; ^4^National Center for Chronic Disease Prevention and Health Promotion/Division of Reproductive Health, Centers for Disease Control and Prevention, Atlanta, GA, United States; ^5^Department of Gynecology and Obstetrics, Emory University School of Medicine, Atlanta, GA, United States; ^6^Hudson Infectious Diseases Associates, Briarcliff Manor, NY, United States; ^7^Division of Infectious Diseases, School of Medicine, Wayne State University, Detroit, MI, United States; ^8^Department of Community Health Systems, School of Nursing, University of California at San Francisco, San Francisco, CA, United States; ^9^The Fenway Institute, Fenway Health, Boston, MA, United States; ^10^Department of Medicine, Beth Israel Deaconess Hospital, Harvard Medical School, Boston, MA, United States

**Keywords:** galectin-1, galectin-3, galectin-9, bacterial vaginosis, *Trichomonas vaginalis*, HPV, endosymbiont viruses, *Prevotella bivia*

## Abstract

Co-infections with sexually transmittable pathogens are common and more likely in women with disturbed vaginal bacteriome. Among those pathogens, the protozoan parasite *Trichomonas vaginalis* (TV) is most common after accounting for the highly persistent DNA viruses human papillomavirus (HPV) and genital herpes. The parasitic infection often concurs with the dysbiotic syndrome diagnosed as bacterial vaginosis (BV) and both are associated with risks of superimposed viral infections. Yet, the mechanisms of microbial synergisms in evading host immunity remain elusive. We present clinical and experimental evidence for a new role of galectins, glycan-sensing family of proteins, in mixed infections. We assessed participants of the HIV Epidemiology Research Study (HERS) at each of their incident TV visits (223 case visits) matched to controls who remained TV-negative throughout the study. Matching criteria included age, race, BV (by Nugent score), HIV status, hysterectomy, and contraceptive use. Non-matched variables included BV status at 6 months before the matched visit, and variables examined at baseline, within 6 months of and/or at the matched visit e.g. HSV-2, HPV, and relevant laboratory and socio-demographic parameters. Conditional logistic regression models using generalized estimating equations calculated odds ratios (OR) for incident TV occurrence with each log_10_ unit higher cervicovaginal concentration of galectins and cytokines. Incident TV was associated with higher levels of galectin-1, galectin-9, IL-1β and chemokines (ORs 1.53 to 2.91, p <0.001). Galectin-9, IL-1β and chemokines were up and galectin-3 down in TV cases with BV or intermediate Nugent *versus* normal Nugent scores (p <0.001). Galectin-9, IL-1β and chemokines were up in TV-HIV and down in TV-HPV co-infections. *In-vitro*, TV synergized with its endosymbiont *Trichomonasvirus* (TVV) and BV bacteria to upregulate galectin-1, galectin-9, and inflammatory cytokines. The BV-bacterium *Prevotella bivia* alone and together with TV downregulated galectin-3 and synergistically upregulated galectin-1, galectin-9 and IL-1β, mirroring the clinical findings of mixed TV–BV infections. *P. bivia* also downregulated TVV+TV-induced anti-viral response e.g. IP-10 and RANTES, providing a mechanism for conducing viral persistence in TV-BV co-infections. Collectively, the experimental and clinical data suggest that galectin-mediated immunity may be dysregulated and exploited by viral–protozoan–bacterial synergisms exacerbating inflammatory complications from dysbiosis and sexually transmitted infections.

## Introduction

Co-infections with taxonomically diverse sexually transmittable pathogens are common and more likely in women with disturbed vaginal bacterial communities (Onderdonk et al., 2016; Brown and Drexler, 2020). Among those pathogens, the extracellular protozoan parasite *Trichomonas vaginalis* (TV) is most common after accounting for human papillomavirus (HPV) and genital herpes (Workowski, 2015). TV accounts for close to half of the annual incidence of nearly half-a-billion sexually transmitted infections (STIs) according to WHO estimates (WHO, 2012; Rowley et al., 2019). Adding to the public health care burden, TV is associated with adverse pregnancy outcomes, high-risk HPV genotypes and cancer (Fichorova, 2009; Yang et al., 2020), as well as with HIV acquisition (Cu-Uvin et al., 1999; Van Der Pol et al., 2008) and transmission (Kissinger et al., 2009). Bacterial vaginosis (BV), a common syndrome of disturbed vaginal microbiota, goes hand-in-hand with TV infection (Onderdonk et al., 2016) and is also associated with HIV acquisition (Atashili et al., 2008), shedding and transmission (Low et al., 2014) and with HPV infection (Oh et al., 2015; Yang et al., 2018; Liang et al., 2019; Brusselaers et al., 2019; Norenhag et al., 2019) and lower rates of HPV clearance (King et al., 2011). BV affects over 21 million (~30%) women of child-bearing age in the US alone (CDC, 2020). Mixed TV-BV infections are common (Brotman et al., 2010), and in HIV-infected women they can dramatically increase HIV shedding in the genital tract, with adjusted odds ratios (OR) as high as 18.63 (95% CI 6.71–51.72) when comparing women with TV and BV to those with neither (Fastring et al., 2014).

Despite the strong epidemiologic evidence for protozoan–bacterial–viral synergisms in evading host immunity in the genital tract mucosa, the molecular mechanisms facilitating coinfections remain largely unknown (Malla et al., 2014). To gain insights into innate immunity breakdown by microbial synergisms, we conducted a nested case-control study utilizing participants from the large HIV Epidemiology Research Study (HERS), which enrolled 1,310 US reproductive age women and followed them over 7 years collecting comprehensive information on STI acquisition and relevant laboratory and socio-demographic variables (Cu-Uvin et al., 1999; Mayer et al., 2003; Tohill et al., 2004; King et al., 2011). We turned our attention to the galectin family of glycan-binding proteins (Sato et al., 2009) as innate immunity mediators yet underexplored in the human cervicovaginal environment despite growing experimental evidence of their involvement in HIV (Lanteri et al., 2003; Ouellet et al., 2005; Mercier et al., 2008; Sato et al., 2012) and TV pathogenesis (Okumura et al., 2008; Fichorova et al., 2016; Heiss et al., 2016). We tested the hypothesis that TV and mixed TV–BV and protozoan–viral (TV-HIV, TV-HPV) infections will be associated with specific patterns of altered galectin levels, which in turn would correlate with altered mediators of innate immunity, e.g. cytokines and chemokines in the cervicovaginal secretions. For the first time in this study we investigated experimentally whether concurrent exposure to TV and BV bacteria dysregulates galectin expression and whether the host–protozoan–bacterial interactions are modified by the protozoan viral endosymbiont *Trichomonasvirus* (TVV), which is commonly carried by the vaginal isolates of the parasite (Fichorova et al., 2017).

## Materials and Methods

### The HERS Cohort

The HERS cohort was started in 1992 before highly active antiretroviral therapy (HAART) became available. A total of 871 HIV-infected women and 439 high-risk HIV-uninfected women between the ages of 16–55 years were enrolled in four U.S. urban centers (Cu-Uvin et al., 1999; Mayer et al., 2003; Tohill et al., 2004). Women with a clinical diagnosis of AIDS were ineligible for enrollment. Women were followed prospectively with comprehensive clinical and laboratory examination every 6 months for up to 7 years. At each visit, a complete pelvic exam was performed that included collection of cervicovaginal lavage (CVL) and vaginal swabs. Polymorphonuclear (PMNs) and mononuclear (MNC) white blood cells (WBC) were enumerated per five oil-immersion microscopy fields. TV, *Neisseria gonorrhea*, *Chlamydia trachomatis*, HSV-2 and HPV were diagnosed as described before (Smith et al., 1997). The Nugent Gram-staining scoring system was used to define normal (score 0–3), altered (score of 4–6) and BV microbiota (score 7–10). A strength of the study was that the Nugent scoring for all samples was performed in a single well-qualified central laboratory (Dr. J. Sobel’s laboratory at Wayne State University, Detroit, MI). Pregnancies, but not breastfeeding, were recorded. As part of a structured interview, women were asked to identify themselves as currently using pill/oral contraceptive, Norplant, or Depo-Provera; if they answered “yes” to any of these, they were listed as taking hormonal contraceptives. Socioeconomic status was measured at enrollment by monthly income, source of income, education, employment, and health insurance coverage and type.

### Matched Case–Control Nested Study Of Incident TV Infection

We defined cases as all HERS participants with documented acquisition of TV who had a CVL collected at a TV+ visit (confirmed by culture), following a prior 6-month visit at which all tests for TV were negative. Each of these TV positive visits post a TV negative visit is referred to as “incident TV infection”. Some women transitioned from TV negative to TV positive status more than once. All such episodes were captured. Women who were negative in all tests for TV recorded throughout study follow-up served as controls. Each incident TV visit was randomly matched 1:1 to a visit by a control participant by race (White, Hispanic/Latina, Black/African American, Native American, Asian) and by the following visit-specific matching criteria: age (within 10 years), HIV status (positive/negative), BV by Nugent categorization, hormonal contraceptive use, and hysterectomy status. We were able to identify 169 women with 223 incident TV infections and 147 control women with 223 matched TV-negative visits. We compared the women selected for the nested case–control study to the overall HERS cohort by all matched variables as well as a number of unmatched socio-behavioral and clinical laboratory parameters. The case–control sample was found to be well-representative of the overall cohort, which provided confidence for the generalizability of the nested case–control findings to the overall study population (**Supplementary Tables 1**, **2**).

### *In-Vitro* Infection Model

#### Isogenic TV Strains

A TV isolate that carries the endosymbiont *Trichomonasvirus* (strain 347v+) and its isogenic derivative strain (347v−) cured from the virus were obtained from Dr. John Alderete (Washington State University) (Provenzano et al., 1997). The status of *Trichomonasvirus* (TVV) infection of each isolate was confirmed as described and reported (Fichorova et al., 2012). Parasites were cultured in modified Diamond’s medium supplemented with 10% heat-inactivated horse serum (HyClone Laboratory) and iron, as reported earlier (Gilbert et al., 2000).

#### Vaginal Bacteria

*Lactobacillus gasseri*, *L. crispatus*, *L. jensenii*, *Gardnerella vaginalis* and *Prevotella bivia* were originally isolated by vaginal swabs from women participating in various vaginal microbiota research studies (Onderdonk et al., 1987; Delaney and Onderdonk, 2001). These isolates were identified using phenotypic characteristics and established criteria (Manual of Clinical Microbiology, Washington (DC): ASM Press; 1995), and identification was confirmed using the Microbial Identification System for long chain fatty acid analysis (MIDI Inc., Newark, DE). *Atopobium vaginae* (ATCC BAA-55) was acquired from the American Type Culture Collection. *L. crispatus* and *L. gasseri* were chosen as common homeostatic *Lactobacillus* species representative of the healthy vaginal microbiota that are non-inflammatory in contrast of the most common BV associated bacteria *P. bivia*, *G. vaginalis* and *A. vaginae* (Fichorova et al., 2013; Anahtar et al., 2015). Although also commonly found in the human vagina, *L. iners* was not included in our experimental homeostatic *Lactobacillus* panel because it has been associated both epidemiologically and causally with disturbed immune homeostasis and vaginal inflammation (Anahtar et al., 2015).

#### Human Epithelial Cell Lines

Immortalized cell lines, originating from normal human vagina (Vk2/E6E7), uterine endocervix (End1/E6E7) and ectocervix (Ect1/E6E7) (Fichorova et al., 1997) were cultured (Fichorova et al., 2011) in antibiotic-free keratinocyte serum-free medium (KSFM), supplemented with 50 μg/ml bovine pituitary extract, 0.1 ng/ml epidermal growth factor (Invitrogen, Carlsbad, CA), and 0.4 mM CaCl_2_ (Fisher Scientific, Pittsburgh, PA). These cell lines have been established as a physiologically relevant *in-vitro* model for the study of *TV* pathogenesis by multiple investigators (Bastida-Corcuera et al., 2005; Fichorova et al., 2006; Okumura et al., 2008; Singh et al., 2009; Lustig et al., 2013; Jain et al., 2014) and have been extensively compared to their primary tissues of origin and to primary organotypic cultures showing no significant differences in responses to *TV* parasites as well as other innate immunity ligands (Fichorova et al., 1997; Fichorova and Anderson, 1999; Fichorova et al., 2002; Fichorova et al., 2004; Canny et al., 2006; Fichorova et al., 2006; Trifonova et al., 2009; Fichorova et al., 2011; Fichorova et al., 2012; Fichorova et al., 2013). The epithelial cell lines can be obtained from ATCC (ATCC^®^ CRL 2616, ATCC^®^ CRL 2615, and ATCC^®^ CRL 2614) or from Dr. Raina Fichorova.

#### Co-Infection Model

TV–BV bacteria co-infection was conducted as previously described (Fichorova et al., 2013). In brief, vaginal epithelial cells grown to confluency were first colonized with bacteria for 24 h followed by removal of cell culture supernatants along with non-adherent bacteria. The colonized epithelial cells were then incubated for 24 h with cell culture medium control, TV 347v+ or TV 347v−. After 24 h incubation under conditions mimicking the vaginal microenvironment (Mitsubishi AnaeroPack, Fisher), cell culture supernatants were collected for assessment of galectin and cytokine levels while cells were harvested for viability assessment by Trypan blue.

### Immunoassays

We measured simultaneously protein levels of galectin-1, -3 and -9 and the following markers of cervicovaginal inflammation; interleukin (IL)-1β, a major cytokine initiator and product of inflammation, and the chemokines IL-8 (CXCL8), interferon gamma-induced protein (IP)-10 (CXCL10), monocyte chemotactic protein (MCP)-1 (CCL2), macrophage inflammatory protein (MIP)-1β (CCL4), RANTES (regulated on activation, normal T cell expressed and secreted) (CCL5), and MIP-3α (CCL19). All ten proteins were quantified in undiluted CVL and cell culture supernatants (stored frozen at −80°C) using a custom-designed multiplex electrochemiluminescence (ECL) immunoassay, Sector Imager 2400 and Discovery Workbench Software (Meso Scale Discovery MSD, Gaithersburg, MD). Validated by comparisons with traditional ELISA (Fichorova et al., 2006; Fichorova et al., 2008), the MSD ECL platform has high clinical content validity (Fichorova et al., 2011). All samples were run in duplicate. All immune mediators were well-detectable within assay linearity in the CVL samples (**Supplementary Table 3**). Galectin-3 was detectable at >33 pg/ml in all CVLs, galectin-9 was detectable at >16 pg/ml in 99% of CVLs, and galectin-1 was detectable at >137 pg/ml in 92% of CVLs.

### Statistics

The subset of women in the HERS cohort selected for the case-control study were compared to those not in this study using t-tests and Fisher Exact tests (**Supplementary Tables 1**, **2**). All immune mediators were log_10_-transformed prior to analysis, and values below the lower limit of detection were set to the lower limit. Due to the use of matched data in this study, odds ratios (OR) are used when comparing the cases and controls. ORs and 95% confidence intervals (CI) for each 1 log_10_ unit higher concentration of the immune mediator with incident *T. vaginalis* (TV) were calculated by conditional logistic regression. To account for potential correlation among multiple longitudinal visits from the same woman, we fitted our conditional logistic regression models using generalized estimating equations (GEE) and employed robust standard errors throughout the analysis. This approach allows for consistent parameter estimation even when the correlation structure is incorrectly or incompletely specified (Liang and Zeger, 1986). Using separate models for each covariate to avoid collinearity, we also used conditional logistic regression to examine the OR associations between log unit higher immune mediator levels and incident TV when visits were stratified on BV, HIV, and other matched and non-matched variables, as well as to compare ORs within levels of these variables.

Correlations between the log_10_-transformed concentrations of immune mediators simultaneously measured in CVLs from all visits were calculated using Pearson’s product moment correlation.

Within the TV positive subgroup only, we used linear regression to evaluate the association between levels of the inflammatory markers as a continuous outcome and several covariates. Again, to account for a possible correlation between multiple visits within a woman, we used generalized estimating questions and employed robust standard errors to construct 95% confidence intervals and calculate p-values (Liang and Zeger, 1986). Covariates examined within the TV positive group were the matched variables as well as unmatched variables that appeared to differ between the TV positive and TV negative women (**Supplementary Tables 1**, **2**) as well as between TV positive and TV negative visits (**Supplementary Table 4**), including Nugent score categories, HIV, HPV, and HSV status, presence of genital tract WBC, smoking and alcohol use. The latter analysis was not done for the control visits, since they were selected to be similar to the matched incident-TV visits on confounders and therefore we could not consider them representative of the broader population of any TV-negative women.

## Results

### Incident TV Is Associated With Higher Cervicovaginal Levels Of Galectin-1 And -9, Which Correlate With Mediators Of Inflammation

Geometric means of galectin-1, galectin-9, IL-1β and all chemokines were higher in women with incident TV compared to matched control visits (**Table 1**). Each log_10_ increase in biomarker concentration was associated with higher odds for incident TV, with the largest ORs seen for increases in galectin-9 (OR = 2.91, 95% CI 2.14–3.97), IL-8 (OR = 2.67, 95% CI 1.99–3.59), IL-1β (OR = 2.56, 95% CI 1.95–3.37), IP-10 (OR = 2.33, 95% CI 1.51–3.60), and galectin-1 (OR = 1.84, 95% CI 1.33–2.55) (p <0.001) (**Table 1**).

**Table 1:** Association between TV positive status and cervicovaginal levels of immune mediators assessed in 223 case and 223 control visits.

Biomarker	Median (IQR) of geometric means in pg/ml		OR (95% CI)	
	TV Positive (n = 223 visits)	TV Negative (n = 223 visits)	All Races (n = 446 visits)	Black Race Only (n = 400 visits)
Galectin-1	1,433 (611–3,850)	871 (309–2,697)	1.84 (1.33, 2.55)***	1.72 (1.20, 2.45)**
Galectin-3	4,340 (2,507–6,361)	4,957 (3,258–7,189)	0.54 (0.29, 1.03)	0.52 (0.27, 1.01)
Galectin-9	2,201 (641–8,451)	598 (196–2,087)	2.91 (2.14, 3.97)***	2.82 (2.03, 3.91)***
IL-1β	63 (14–262)	10 (3–63)	2.56 (1.95, 3.37)***	2.55 (1.90, 3.43)***
IL-8	1,133 (410–5,796)	259 (93–1,049)	2.67 (1.99, 3.59)***	2.61 (1.91, 3.57)***
IP-10	291 (96–789)	157 (58–398)	2.33 (1.51, 3.60)***	2.17 (1.38, 3.42)***
MCP-1	8 (3–29)	5 (2–19)	1.53 (1.16, 2.00)**	1.47 (1.11, 1.96)**
MIP-1β	17 (8–50)	10 (7–40)	1.67 (1.13, 2.46)**	1.49 (0.99, 2.24)
RANTES	11 (4-41)	3 (1–19)	1.72 (1.30, 2.28)***	1.64 (1.23, 2.20)***
MIP-3α	200 (96–496)	127 (50–321)	1.77 (1.21, 2.60)**	1.75 (1.17, 2.61)**

Median and inter-quartile ranges (IQR) of back-transformed geometric means of concentrations of immune mediators and odds ratios (OR) and 95% confidence intervals (CI) for incident *T. vaginalis* (TV) with each 1 log_10_ unit higher level of immune mediator are calculated from conditional logistic regression models fitted using generalized estimating equations. Significantly different ORs are bolded and indicated by **p < 0.01 and ***p < 0.001.

The size of OR and 95% CI were similar in Black women who represented the majority of the women with incident TV selected for our case-control sample (148/169, 88%) (**Table 1**) as well as the majority of overall HERS cohort participants (736/1310, 59%) and women infected with TV at baseline and throughout the study (445/566, 79%) (**Supplementary Tables 1**, **2**).

A strong positive correlation (Pearson correlation coefficient ≥0.5) was observed between galectin-1 and galectin-9, between galectin-1 and all cytokines and chemokines, and between galectin-9 and IL-1β, IL-8, MIP-1β, and RANTES, but not between galectin-3 and any of those immune mediators (**Table 2**).

**Table 2:** Pearson correlation between cervicovaginal levels of immune mediators in all 446 visits.

Immune Mediator	Galectin-1	Galectin-3	Galectin-9	IL-1β	IL-8 (CXCL8)	IP-10 (CXCL10)	MCP-1 (CCL2)	MIP-1β (CCL4)	MIP-3α (CCL20)	RANTES (CCL5)
Galectin-1	1		0.6	0.6	0.7	0.6	0.6	0.8	0.6	0.8
Galectin-3		1								
Galectin-9	0.6		1	0.8	0.8			0.6		0.7
IL-1β	0.6		0.8	1	0.9			0.6		0.6
IL-8	0.7		0.8	0.9	1			0.6		0.6
IP-10	0.6					1	0.6			0.5
MCP-1	0.6					0.6	1			0.6
MIP-1β	0.8		0.6	0.6	0.6			1		0.7
MIP-3α	0.6								1	
RANTES	0.8		0.7	0.6	0.6	0.5	0.6	0.7		1

Only coefficients ≥0.5 shown. In gray shade; the black shading denotes each protein compared to itself.

### Higher Galectin-1 and -9 Are Associated With Incident TV Co-Infections With BV, HIV, HPV or HSV, While Galectin-3 Is Negatively Associated With Incident TV Co-Infections With HIV And Normal Bacterial Flora

**Figure 1:**
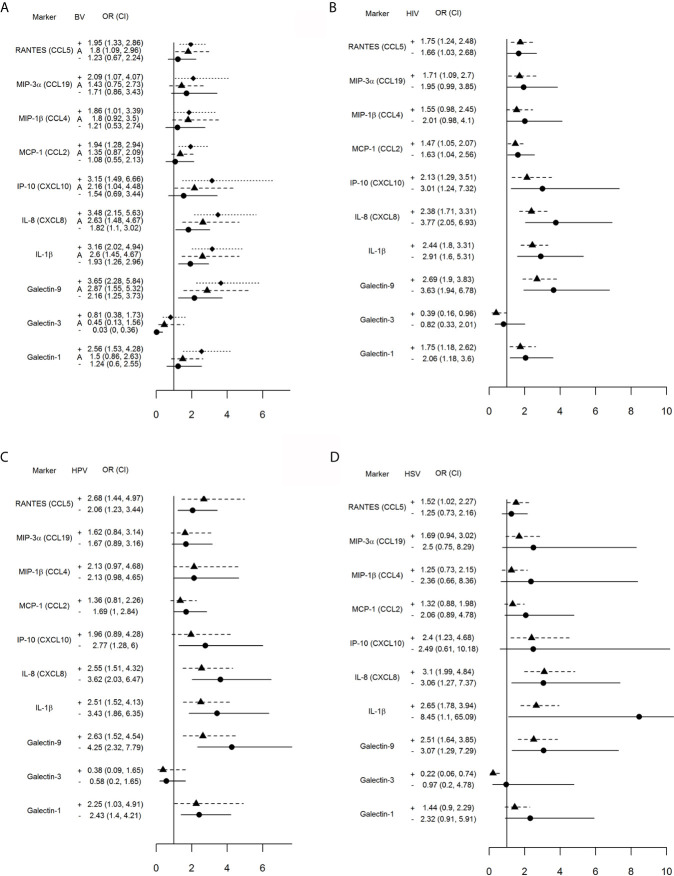
Impact of vaginal microbiota disturbance **(A)**, HIV **(B)**, HPV **(C)** and genital herpes **(D)** on odds ratios (OR) and 95% confidence intervals (CI) for incident TV with each one log_10_ unit increase in immune mediator levels calculated for all 446 visits stratified by: **(A)** BV[+]: Nugent score = 7–10 (square symbol, dotted line), BV[–]: Nugent score 0–3 (circle symbol, solid line), and A[+] (triangle symbol, dashed line): altered microbiota, Nugent score = 4–6; **(B)** HIV[+] (triangle, dashed line) and [–] status (circle, solid line); **(C)** HPV [+] (triangle, dashed line) or [–] status (circle, solid line), and **(D)** HSV [+] (triangle, dashed line) and [–] (circle, solid line) status. The vertical line is set at 1.0, representing an OR of 1. Any 95% CI >1 or <1 indicates significant positive or respectively negative assocaition between immune mediator levels and TV positive status within each stratum (+, A, or – in **A** and + or – in **B–D**).

Separate conditional logistic regression models examined the association between each one log_10_ higher level of immune mediator and incident TV when stratified by co-infection status at the case-control matched visit (**Figures 1A–D**).

An interaction was observed between TV and concurrent abnormal vaginal microbiota (**Figure 1A**). Incident TV was associated with higher RANTES, MIP-3α, MIP-1β, MCP-1 and galectin-1 only when BV-positive, and higher IP-10—only when positive for BV or Nugent score 4–6. Higher galectin-9, IL-1β and IL-8 were significantly associated with incident TV in each Nugent categories; however, the 95% CI for the OR shifted closer to 1.0 within each Nugent category shift closer to normal vaginal flora. The relationship between galectin-3 and incident TV was inverted, with significant negative association only when normal microbiota was present.

The concurrent HIV positive status had less impact on the association between incident TV and immune mediators with the most notable exception of galectin-3 which was inversely associated with incident TV in the HIV-positive but not HIV-negative visits (**Figure 1B**).

Higher levels of galectin-9, IL-1β, IL-8, RANTES and galectin-1 were significantly associated with incident TV in both HPV-positive and -negative visits while higher IP-10 was associated with incident TV in HPV negative visits only (**Figure 1C**).

Higher levels of galectin-9, IL-1β and IL-8 were significantly associated with incident TV in both HSV negative and HSV positive visits; however, higher levels of RANTES and IP-10 and lower levels of galectin-3 were associated with incident TV in the HSV positive visits only (**Figure 1D**).

### Co-Infections, Cervicovaginal Leukocytes, Hysterectomy, And Socio-Behavioral Variables Affect Levels Of Galectins And Inflammatory Mediators At Incident TV

To identify factors that may affect levels of immune mediators and galectins associated with TV incidence, we examined non-matched socio-behavioral and clinical laboratory parameters at baseline, 6 months prior to matched visit and at the matched 446 visits comparing cases to controls (**Supplementary Table 4**).

At baseline, incident TV cases were less likely to have above high school education (OR = 0.47; CI = 0.27–0.80, p = 0.006), be employed (OR = 0.41, 95% CI0.26–0.67, p <0.001), be on Medicaid (OR = 0.64; 95% CI 0.43–0.95, p = 0.026) and having no sex *vs* using a condom all the time with a male partner (OR = 0.41, 95% CI 0.23–0.75), p = 0.004). There was no significant difference by HSV-2 serology at baseline.

When tested for HPV at 6 months prior to the matched visit, the TV incident visit was most likely to have a switch from HPV-positive to -negative status (OR = 3.35; 95% CI 1.62–6.95, p = 0.001), followed by newly acquired HPV (OR = 2.74, 95% CI 1.27–5.89, p = 0.010) and a persistent HPV-positive test (OR = 1.95; 95% CI 1.16–3.26, p = 0.011).

Socio-behavioral covariates more likely to be reported by incident TV cases than controls during the 6 months prior to the matched visit included cigarette smoking (OR = 2.52; 95% CI 1.58–4.01, p <0.001), alcohol (OR = 2.29; 95% CI 1.53–3.44, p <0.001) and illicit drug use (OR = 3.11; 95% CI 2.03–4.76, p <0.001), one male partner *versus* none (OR = 1.92, 95% CI 1.15–3.22, p = 0.013) and 3+ male partners *versus* none (OR = 2.38, 95% CI 1.09–5.22, p = 0.03).

At the matched visit, incident TV was more likely to test positive *versus* negative for cervicovaginal MNCs (OR = 2.33; 95% CI 1.27–4.28, p = 0.006), positive for both PMNs and MNCs *versus* PMNs only (OR 2.45; 95% CI 1.22–4.89, p = 0.011), and positive *versus* negative for HPV (OR = 1.63; 95% CI 1.07–2.48, p = 0.023). Incident TV and controls did not differ by non-matched HIV variables, e.g. CD4+ T cell counts, HIV plasma load and antiretroviral subcategories.

**Figure 2:**
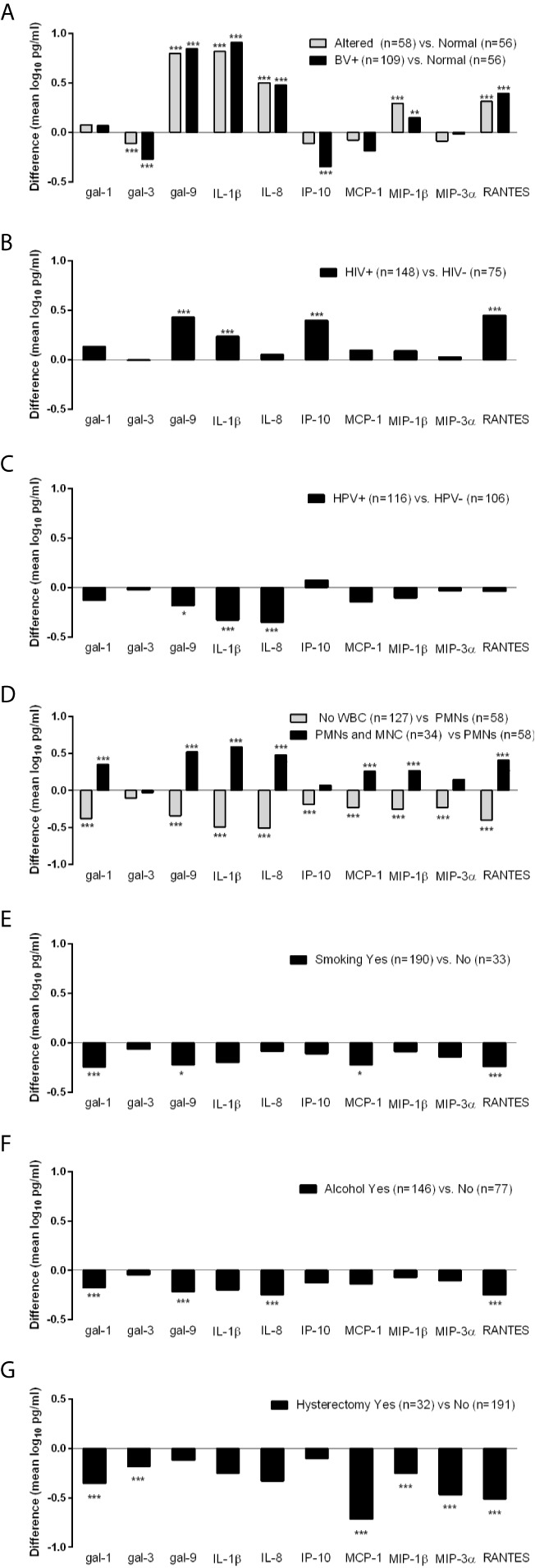
Differences in levels of immune mediators (mean log_10_ pg/ml) measured at the incident TV visits stratified by BV **(A)**, HIV **(B)**, HPV **(C)**, presence of white blood cells (WBC) including polymorphonuclear (PMNs) and mononuclear (MNC) cells **(D)** and hysterectomy **(G)** status at the time of immunologic assessment, and by cigarette smoking **(E)** and alcohol consumption **(F)** during the 6 months preceding the immunologic assessment. Differences were assessed using ordinary least squares linear regression (ANOVA) with *p < 0.05, **p < 0.01, and ***p < 0.001.

To further identify factors that may be driving the levels of galectins and inflammatory mediators among the TV incident visits only, we performed regression analysis with focus on matched variables (Nugent score, HIV status and hysterectomy) and non-matched variables that differed at the matched and 6-month prior visits. Only factors showing at least one significant association with the immune mediators are shown in **Figure 2**.

Differences in galectin levels were observed when TV incident visits were stratified by Nugent score into three strata: BV positive (Nugent >7), intermediate/altered microbiota (Nugent = 4–7) and normal microbiota (Nugent <4) (p <0.01 and <0.001, **Figure 2A**). In comparison to visits when normal microbiota was found, visits diagnosed with BV and altered microbiota had lower levels of galectin-3 and higher levels of galectin 9, IL-1β, IL-8, MIP-1β and RANTES. In addition, those with BV had lower levels of IP-10.

When compared by HIV status, TV-HIV coinfections showed higher levels of galectin-9, IL-1β, IP-10 and RANTES compared to HIV negative visits (p <0.001, **Figure 2B**). TV-HPV co-infections HPV in contrast showed lower levels of galectin-9 (p <0.05), IL-1β and IL-8 (p <0.001) (**Figure 2C**).

The co-presence of PMNs and MNCs compared to PMNs alone was associated with significantly higher levels of all immune mediators (p <0.001) except galectin-3, IP-10 and MIP-3α. The absence of any WBC was associated with significantly lower levels of all immune mediators including galectins-1 and -9 (p <0.001) but not galectin-3 which did not reach significance. These data suggested that WBC contribute to higher levels of galectin-1 and galectin-9 in incident TV (**Figure 2D**).

Smoking and alcohol use were similarly associated with lower levels of multiple mediators e.g. galectin-1 (p <0.001), galectin-9 (p <0.05 in smokers and p <0.001 if alcohol users) and RANTES (p <0.001) (**Figures 2E, F**), suggesting contribution to a suppressed immunity.

Interestingly, incident TV following hysterectomy showed lower levels of galectin-1, galectin-3, and chemokines MCP-1, MIP-1β, MIP-3α, and RANTES (p <0.001), suggesting a contribution of the upper reproductive tract to immune responses in TV infection (**Figure 2G**).

### Bacterial–Protozoan–Viral Synergisms Upregulate Galectin-1, -9 And Interleukin-1β, Downregulate Galectin-3 And Selectively Alter Chemokine Expression

**Figure 3:**
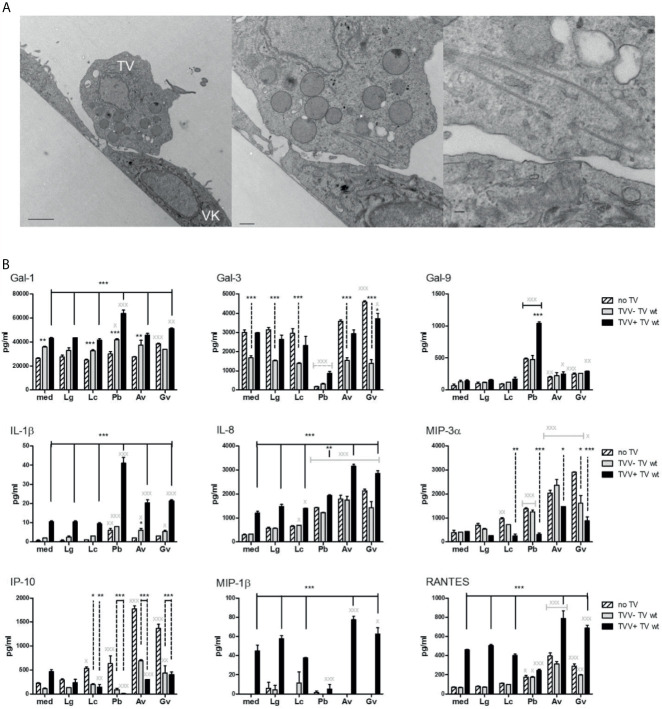
Polymicrobial regulation of galectins and inflammatory mediators in a human vaginal infections model. **(A)** *T. vaginalis* (TV) adherent to human vaginal epithelial cells illustrated by transmission electron microscopy. **(B)** Levels of immune mediators measured in the vaginal epithelial supernatants after 24 h colonization with bacteria followed by 24 h exposure to TV. The epithelial cells were first colonized with *Lactobacillus* species characteristic for the healthy vaginal microbiota (*L. gasseri* = Lg and *L. crispatus* = Lc) or BV bacteria (*P. bivia* = Pb, *A. vaginae* = Av and *G. vaginalis* = Gv). Then the bacteria-colonized epithelial cells were infected with TV protozoa harboring *Trichomonas vaginalis* virus (TVV+) or a derivative TV strain that was cured from the virus (TVV−). Bars represent mean and SD from triplicate cultures in one of three experiments. P values are from ANOVA with Bonferroni multiple comparison test. ^X,^*p < 0.05; ^XX,^**p < 0.01; ^XXX,^***p < 0.001, different from medium (med) control.

To test the causality underlying epidemiologic TV–BV interactions, we applied an established experimental human infection model (**Figure 3A**). In order to investigate the effect of mixed infection, we infected bacteria-colonized epithelial cells with TVV-positive parasites and their TVV-cured isogenic counterparts (Provenzano et al., 1997).

In the absence of bacteria, the TVV-cured TV caused little change in the cytokine/chemokine levels, but significantly upregulated galectin-1 (p <0.01) and downregulated galectin-3 (p <0.001) (**Figure 3B**), consistent with prior findings with naturally occurring TVV-negative TV (Fichorova et al., 2016). In contrast, the naturally occurring TVV-positive isolate upregulated most immune mediators (p <0.001) except galectin-3, galectin-9, and MIP-3α (**Figure 3B**).

In the absence of TV, *Lactobacillus* colonization caused no change with the exception of upregulated MIP-3α (p <0.01) and IP-10 (p <0.05) by *L. crispatus* (**Figure 3B**). In contrast, colonization by BV bacteria selectively upregulated IL-1β and dysregulated galectins and chemokines (**Figure 3B**). All three BV bacteria upregulated galectin-9 (p <0.01). In addition, *G. vaginalis* significantly upregulated galectin-1 and -3 (P <0.001), while *P. bivia* almost completely blocked galectin-3 expression (p <0.001). All three BV bacteria upregulated IL-8, MIP-3α, IP-10 (p <0.001) and RANTES (p <0.05, *P. bivia*, and p<0.001, G. *vaginalis* and *A. vaginae*), whereas *P. bivia* also upregulated IL-1β (p <0.01).

The presence of bacteria modified the effects of both virus-infected and cured protozoa and vice versa (**Figure 3B**). The co-infection with TVV+TV and *P. bivia* synergistically upregulated galectin-1, -9 and IL-1β (p <0.001). Galectin-3 levels were lower in TV-*P. bivia* co-infection compared to TV infection alone, regardless of TVV status (p <0.001). *P. bivia* abolished the TVV+TV-induced upregulation of MIP-1β and IP-10 (p <0.001). TVV+TV downregulated the stimulatory effect of all bacteria on MIP-3α and both TVV+ and TVV−TV downregulated their stimulatory effect on IP-10 (p <0.001).

## Discussion

This study is the first to identify galectins as a molecular basis for protozoan–viral–bacterial synergisms at the mucosal interface.

In our cohort, higher galectin-1 and galectin-9 levels correlated with markers of cervicovaginal inflammation, marked by higher levels of IL-1β and chemokines e.g. IL-8. In TV infected women they were associated with vaginal dysbiosis/BV and with presence of monocytes in the mucosal secretions, which are additional risk factors for HIV acquisition and transmission (Alfano and Poli, 2002). Prior clinical studies have shown higher levels of IL-1β and IL-8 in women with concurrent BV and TV as compared to women with neither infection (Cauci and Culhane, 2007); however, we show for the first time higher levels of galectin-1, RANTES, and IP-10 to be associated with incident TV in women with BV only.

We provide new experimental evidence that galectin-1 and galectin-9 are synergistically upregulated in host epithelial cells by virus-infected TV and the BV pathobiont *P. bivia*, emphasizing the need to study the individual complements of the disturbed vaginal microbiome as drivers of immune imbalances. Our data suggest molecular mechanisms by which BV and particularly *P. bivia* may be driving susceptibility to persistent TV infection. By upregulating epithelial galectin-1 expression *P. bivia* may contribute to the amplified galectin-1 levels in the natural TV-BV coinfection and thereby facilitate the adherence of TV to epithelial cells (Okumura et al., 2008), which is mediated by galectin-1 binding to the protozoan surface lipophosphoglycan (LPG) (Fichorova et al., 2016).

The increased levels of galectin-1 and -9 also offer a molecular basis for bacterial–protozoan synergism with HIV and other viruses. Galectin-1 facilitates HIV attachment to macrophages (Ouellet et al., 2005; Mercier et al., 2008), and in turn, HIV-1 amplifies this effect *via* altering cell surface glycosylation (Lanteri et al., 2003). Galectin-9 also promotes HIV entry into host cells (Bi et al., 2011). Higher levels of galectin-9 may contribute to CD8+ T cell exhaustion thorough biding to Tim-3 (Merani et al., 2015). Knocking down galectin-9 in mouse models improved clearance of HSV infections (Merani et al., 2015) and we saw trend of higher galectin-9 levels in the TV-HSV co-infection but this association did not reach significance.

Individual BV-associated organisms upregulated IL-1β, IL-8, MIP-3α, IP-10 and RANTES in our *in-vitro* model and our experiments with isogenic virus-positive and virus-negative TV parasites supported previous findings of bacterial synergisms with soluble LPG and molecular domain and with cell-free endosymbiont protozoan viruses, leading to upregulation of IL-1β (*P. bivia*) and IL-8 (*G. vaginalis*, *A. vaginae*) (Fichorova et al., 2013). We have also shown that the genomic dsRNA originating from the protozoan TVV viruses can initiate viral stress response in human vaginal and cervical epithelial cells through TLR3/IRF3 signaling (Fichorova et al., 2012). The TLR3/IRF3 pathway activation can explain the galectin-9 upregulation by TVV as shown with a dsRNA viral mimic in vascular cells (Imaizumi et al., 2007). The stimulation of an anti-viral immune response by TVV+TV alone includes anti-viral immune mediators e.g. IP-10, MIP-1b and RANTES (this study and (Fichorova et al., 2012). However, this potentially protective immune alarming function of the vaginal epithelium was dampened in the presence of TV co-infection with the BV-signature bacterium *P. bivia* providing a plausible mechanism for hampering HPV clearance in TV-BV co-infections. In the HERS study BV but not TV alone was associated with increased odds of prevalent and incident HPV and delayed HPV clearance (King et al., 2011). The strong (over two logs) synergistic upregulation of galectin-9 by TVV+TV and *P. bivia* and higher levels of galectin-9 which we observed in TV–HPV co-infections may provide an additional boost to persistence of high-risk HPV genotypes since galectin-9 expression has been significantly correlated with the presence of HPV type 16 or 18 in cervical cancer tissue (Punt et al., 2015). The innate anti-viral role of galectin-9 secreted in the cervicovaginal secretions and how it may be hijacked by vaginal pathogens is yet to be elucidated. Further studies are needed to investigate galectin-1 and galectin-9 mediated signaling in TV–BV–HPV interactions, to what extend they may be protective and whether our finding of suppressed innate immune responses in mixed HPV–TV infections *versus* TV alone relates to susceptibility to cervical cancer observed in women with TV (Gram et al., 1992; Viikki et al., 2000; Misra and Singh, 2006; Depuydt et al., 2010; Rodriguez-Cerdeira et al., 2012).

Furthermore, our study suggests for a first time a distinct role for galectin-3 which was adversely associated with TV infection both clinically and experimentally. Clinically, we observed that with higher soluble levels of galectin-3 in their cervicovaginal secretions women had significantly lower OR of having incident TV when having normal Nugent score (OR = 0.03, 95% CI = 0, 0.36) or co-infection with HIV (OR 0.39, 95% CI = 0.16–0.96) or HSV (OR = 0.22, 95% CI = 0.06–0.74) (**Figure 1**). These results supported that lower galectin-3 levels are associated with higher likelihood of TV incidence but that this association depends on bacterial and viral co-infection status. We have previously shown that the parasite alone, in the absence of endosymbiont virus and bacteria, depletes extracellular galectin-3 levels, which benefits the parasite survival by suppressing chemokine-driven host immune defenses (Fichorova et al., 2016). We now show experimentally that galectin-3 levels were further synergistically suppressed in TV co-infection with *P. bivia* (**Figure 3**), which provides a mechanism for clinically reduced cervicovaginal galectin-3 levels in TV–BV co-infected women compared to TV alone observed in our study (**Figure 2**). Galectin-3 stimulates innate and adaptive immunity (Rabinovich et al., 2004; Stowell et al., 2008), and thus lowering the extracellular levels of galectin-3 would be expected to decrease the clearance or resistance to both bacterial and protozoan infection. HIV on the other hand hijacks cell-associated galectin-3 by driving its expression *via* the HIV tat protein (Fogel et al., 1999), which appears to supports the initial stage of HIV infection (Schroder et al., 1995) and viral budding (Wang et al., 2014). The TV parasite may not interfere with this initial stage of HIV infection because we have shown that experimentally it decreases extracellular, but not cell-associated, galectin-3 (Fichorova et al., 2016).

The role of *P. bivia* in the polymicrobial syndrome of vaginal immunity breakdown requires special attention. Women with high relative abundance of *P. bivia* in vaginal metagenome analysis were shown to be 19 times more likely to have a pro-inflammatory vaginal cytokine profile and ~13 times more likely to acquire HIV, compared to all others in a South African cohort (Eastment and McClelland, 2018). Moreover, *P. bivia* is the single BV organism linked to HPV gene expression and metastatic cervical cancer (Lam et al., 2018). Our experimental model confirmed the causative role of this Gram-negative anaerobe as a modifier of anti-viral and anti-protozoan host immune response. Further studies are needed to investigate the role of galectin-1, -3. -9 and other galectins in BV–TV–HIV–HPV co-infections and their specific interaction with *P. bivia*.

Lastly, we observed suppressed vaginal immunity, including galectins-1 and -9, in women with incident TV who smoked or consumed alcohol. It is possible that tobacco or alcohol substances have a direct immunosuppressive effect that contributes to the higher risk of incident TV diagnosed in HERS participants reporting their use. Emerging clinical evidence implicates smoking in supporting BV-associated microbial communities, and cessation of smoking was suggested as a plausible pre-requisite for restoring healthy *Lactobacillus* dominance, providing a pathway for a causative effect on vaginal innate immunity (Brotman et al., 2014). Alternatively, smoking and alcohol consumption may be correlates of other risk factors, such as lower socio-economic status, stress and depression capable of acting as immunity modifiers, which we were unable to investigate in our sample. A further limitation of our study is that it included predominantly Black women with or at high risk for HIV and only non-pregnant women and we had limited information on methods of contraception. Thus, we were unable to assess galectins in polymicrobial infections in the context of race, ethnicity and associated effect modifiers, as well as in the context of pregnancy, breastfeeding and hormonal contraceptive use, previously associated with altered cervical immunity in women with BV, TV and viral STIs (Morrison et al., 2014). The results presented here may help assess the power needed for future studies to be conducted in diverse populations to address the role of socio-demographic factors and health disparities in galectin-mediated immunity underlying susceptibility to mixed protozoan–viral–bacterial infections.

## Data Availability Statement

The datasets for this study are publicly available through the Centers for Disease Control and Prevention (CDC) where the HERS database is maintained. A written request reviewed by CDC is required. To obtain details on how to request access contact the corresponding author Dr. Fichorova at rfichorova@bwh.harvard.edu.

## Ethics Statement

The studies involving human participants were reviewed and approved by the Institutional Review Board (IRB) at each participating institution at HERS' four study sites (Bronx, NY; Baltimore, MD; Detroit, MI; and Providence, RI), and at the CDC and Brigham and Women’s Hospital. Written informed consent to participate in this study was provided by the participants' legal guardian/next of kin.

## Author Contributions

RNF conceived the case-control and experimental study and drafted the manuscript. AKD provided the statistical method and analysis. RNF and HSY contributed to immunologic data acquisition. All authors contributed to the data interpretation and provided a critical review and approval of the manuscript.

## Funding

This study was supported by NIH/NIAID grants R01AI079085, 1R56AI091889-01A1 and 1RC1AI086788-01 (RNF).

## Conflict of Interest

The authors declare that the research was conducted in the absence of any commercial or financial relationships that could be construed as a potential conflict of interest.

## Publisher’s Note

All claims expressed in this article are solely those of the authors and do not necessarily represent those of their affiliated organizations, or those of the publisher, the editors and the reviewers. Any product that may be evaluated in this article, or claim that may be made by its manufacturer, is not guaranteed or endorsed by the publisher.

## References

[B1] Alfano M. Poli G. (2002). The Cytokine Network in HIV Infection. Curr. Mol. Med. 2, 677–689. 10.2174/1566524023361925 12462389

[B2] Anahtar M. N. Byrne E. H. Doherty K. E. Bowman B. A. Yamamoto H. S. Soumillon M.. (2015). Cervicovaginal Bacteria Are a Major Modulator of Host Inflammatory Responses in the Female Genital Tract. Immunity 42, 965–976. 10.1016/j.immuni.2015.04.019 25992865 PMC4461369

[B3] Atashili J. Poole C. Ndumbe P. M. Adimora A. A. Smith J. S. (2008). Bacterial Vaginosis and HIV Acquisition: A Meta-Analysis of Published Studies. AIDS 22, 1493–1501. 10.1097/QAD.0b013e3283021a37 18614873 PMC2788489

[B4] Bastida-Corcuera F. D. Okumura C. Y. Colocoussi A. Johnson P. J. (2005). Trichomonas Vaginalis Lipophosphoglycan Mutants Have Reduced Adherence and Cytotoxicity to Human Ectocervical Cells. Eukaryot. Cell 4, 1951–1958. 10.1128/EC.4.11.1951-1958.2005 16278462 PMC1287856

[B5] Bi S. Hong P. W. Lee B. Baum L. G. (2011). Galectin-9 Binding to Cell Surface Protein Disulfide Isomerase Regulates the Redox Environment to Enhance T-Cell Migration and HIV Entry. Proc. Natl. Acad. Sci. U. S. A. 108, 10650–10655. 10.1073/pnas.1017954108 21670307 PMC3127870

[B6] Brotman R. M. He X. Gajer P. Fadrosh D. Sharma E. Mongodin E. F.. (2014). Association Between Cigarette Smoking and the Vaginal Microbiota: A Pilot Study. BMC Infect. Dis. 14, 471–471. 10.1186/1471-2334-14-471 25169082 PMC4161850

[B7] Brotman R. M. Klebanoff M. A. Nansel T. R. Yu K. F. Andrews W. W. Zhang J.. (2010). Bacterial Vaginosis Assessed by Gram Stain and Diminished Colonization Resistance to Incident Gonococcal, Chlamydial, and Trichomonal Genital Infection. J. Infect. Dis. 202, 1907–1915. 10.1086/657320 21067371 PMC3053135

[B8] Brown H. Drexler M. (2020). Improving the Diagnosis of Vulvovaginitis: Perspectives to Align Practice, Guidelines, and Awareness. Popul. Health Manag. 23, S3–S12. 10.1089/pop.2020.0265 32997581 PMC7591372

[B9] Brusselaers N. Shrestha S. van de Wijgert J. Verstraelen H. (2019). Vaginal Dysbiosis and the Risk of Human Papillomavirus and Cervical Cancer: Systematic Review and Meta-Analysis. Am. J. Obstet. Gynecol. 221, 9–18.e18. 10.1016/j.ajog.2018.12.011 30550767

[B10] Canny G. O. Trifonova R. T. Kindelberger D. W. Colgan S. P. Fichorova R. N. (2006). Expression and Function of Bactericidal/Permeability-Increasing Protein in Human Genital Tract Epithelial Cells. J. Infect. Dis. 194, 498–502. 10.1086/505712 16845634

[B11] Cauci S. Culhane J. F. (2007). Modulation of Vaginal Immune Response Among Pregnant Women With Bacterial Vaginosis by Trichomonas Vaginalis, Chlamydia Trachomatis, Neisseria Gonorrhoeae, and Yeast. Am. J. Obstet. Gynecol. 196, 133.e131–133.e137. 10.1016/j.ajog.2006.08.033 17306653

[B12] CDC (2020). Bacterial Vaginosis (BV) Statistics. Available at: https://www.cdc.gov/std/bv/stats.htm.

[B13] Cu-Uvin S. Hogan J. W. Warren D. Klein R. S. Peipert J. Schuman P.. (1999). Prevalence of Lower Genital Tract Infections Among Human Immunodeficiency Virus (HIV)-Seropositive and High-Risk HIV-Seronegative Women. HIV Epidemiology Research Study Group. Clin. Infect. Dis. 29, 1145–1150. 10.1086/313434 10524955

[B14] Delaney M. L. Onderdonk A. B. (2001). Nugent Score Related to Vaginal Culture in Pregnant Women. Obstet. Gynecol. 98, 79–84. 10.1016/S0029-7844(01)402-8 11430961

[B15] Depuydt C. E. Leuridan E. Van Damme P. Bogers J. Vereecken A. J. Donders G. G. (2010). Epidemiology of Trichomonas Vaginalis and Human Papillomavirus Infection Detected by Real-Time PCR in Flanders. Gynecol. Obstet. Invest. 70, 273–280. 10.1159/000314017 21051847

[B16] Eastment M. C. McClelland R. S. (2018). Vaginal Microbiota and Susceptibility to HIV. AIDS 32, 687–698. 10.1097/QAD.0000000000001768 29424773 PMC5957511

[B17] Fastring D. R. Amedee A. Gatski M. Clark R. A. Mena L. A. Levison J.. (2014). Co-Occurrence of Trichomonas Vaginalis and Bacterial Vaginosis and Vaginal Shedding of HIV-1 RNA. Sex Transm. Dis. 41, 173–179. 10.1097/OLQ.0000000000000089 24521723

[B18] Fichorova R. N. (2009). Impact of T. Vaginalis Infection on Innate Immune Responses and Reproductive Outcome. J. Reprod. Immunol. 83, 185–189. 10.1016/j.jri.2009.08.007 19850356 PMC2788009

[B19] Fichorova R. N. Anderson D. J. (1999). Differential Expression of Immunobiological Mediators by Immortalized Human Cervical and Vaginal Epithelial Cells. Biol. Reprod. 60, 508–514. 10.1095/biolreprod60.2.508 9916021

[B20] Fichorova R. N. Bajpai M. Chandra N. Hsiu J. G. Spangler M. Ratnam V.. (2004). Interleukin (IL)-1, IL-6, and IL-8 Predict Mucosal Toxicity of Vaginal Microbicidal Contraceptives. Biol. Reprod. 71, 761–769. 10.1095/biolreprod.104.029603 15128598

[B21] Fichorova R. N. Buck O. R. Yamamoto H. S. Fashemi T. Dawood H. Y. Fashemi B.. (2013). The Villain Team-Up or How Trichomonas Vaginalis and Bacterial Vaginosis Alter Innate Immunity in Concert. Sex Transm. Infect. 89, 460–466. 10.1136/sextrans-2013-051052 23903808 PMC3746192

[B22] Fichorova R. N. Cronin A. O. Lien E. Anderson D. J. Ingalls R. R. (2002). Response to Neisseria Gonorrhoeae by Cervicovaginal Epithelial Cells Occurs in the Absence of Toll-Like Receptor 4-Mediated Signaling. J. Immunol. 168, 2424–2432. 10.4049/jimmunol.168.5.2424 11859134

[B23] Fichorova R. Fraga J. Rappelli P. Fiori P. L. (2017). Trichomonas Vaginalis Infection in Symbiosis With Trichomonasvirus and Mycoplasma. Res. Microbiol. 168, 882–891. 10.1016/j.resmic.2017.03.005 28366838 PMC8130574

[B24] Fichorova R. N. Lee Y. Yamamoto H. S. Takagi Y. Hayes G. R. Goodman R. P.. (2012). Endobiont Viruses Sensed by the Human Host - Beyond Conventional Antiparasitic Therapy. PLoS One 7 (11), e48418. 10.1371/journal.pone.0048418 23144878 PMC3492353

[B25] Fichorova R. N. Onderdonk A. B. Yamamoto H. Delaney M. L. DuBois A. M. Allred E.. (2011). Maternal Microbe-Specific Modulation of Inflammatory Response in Extremely Low-Gestational-Age Newborns. mBio 2, e00280–e00210. 10.1128/mBio.00280-10 PMC3025357 21264056

[B26] Fichorova R. N. Rheinwald J. G. Anderson D. J. (1997). Generation of Papillomavirus-Immortalized Cell Lines From Normal Human Ectocervical, Endocervical, and Vaginal Epithelium That Maintain Expression of Tissue-Specific Differentiation Proteins. Biol. Reprod. 57, 847–855. 10.1095/biolreprod57.4.847 9314589

[B27] Fichorova R. N. Richardson-Harman N. Alfano M. Belec L. Carbonneil C. Chen S.. (2008). Biological and Technical Variables Affecting Immunoassay Recovery of Cytokines From Human Serum and Simulated Vaginal Fluid: A Multicenter Study. Anal. Chem. 80, 4741–4751. 10.1021/ac702628q 18484740 PMC2646866

[B28] Fichorova R. N. Trifonova R. T. Gilbert R. O. Costello C. E. Hayes G. R. Lucas J. J.. (2006). Trichomonas Vaginalis Lipophosphoglycan Triggers a Selective Upregulation of Cytokines by Human Female Reproductive Tract Epithelial Cells. Infect. Immun. 74, 5773–5779. 10.1128/IAI.00631-06 16988255 PMC1594934

[B29] Fichorova R. N. Yamamoto H. S. Delaney M. L. Onderdonk A. B. Doncel G. F. (2011). Novel Vaginal Microflora Colonization Model Providing New Insight Into Microbicide Mechanism of Action. MBio 2, e00168–e00111. 10.1128/mBio.00168-11 22027006 PMC3202752

[B30] Fichorova R. N. Yamamoto H. S. Fashemi T. Foley E. Ryan S. Beatty N.. (2016). Trichomonas Vaginalis Lipophosphoglycan Exploits Binding to Galectin-1 and -3 to Modulate Epithelial Immunity. J. Biol. Chem. 291, 998–1013. 10.1074/jbc.M115.651497 26589797 PMC4705417

[B31] Fogel S. Guittaut M. Legrand A. Monsigny M. Hebert E. (1999). The Tat Protein of HIV-1 Induces Galectin-3 Expression. Glycobiology 9, 383–387. 10.1093/glycob/9.4.383 10089212

[B32] Gilbert R. O. Elia G. Beach D. H. Klaessig S. Singh B. N. (2000). Cytopathogenic Effect of Trichomonas Vaginalis on Human Vaginal Epithelial Cells Cultured *In Vitro* . Infect. Immun. 68, 4200–4206. 10.1128/IAI.68.7.4200-4206.2000 10858237 PMC101726

[B33] Gram I. T. Macaluso M. Churchill J. Stalsberg H. (1992). Trichomonas Vaginalis (TV) and Human Papillomavirus (HPV) Infection and the Incidence of Cervical Intraepithelial Neoplasia (CIN) Grade III. Cancer Causes Control 3, 231–236. 10.1007/BF00124256 1319218

[B34] Heiss C. Wang Z. Black I. Azadi P. Fichorova R. N. Singh B. N. (2016). Novel Structural Features of the Immunocompetent Ceramide Phospho-Inositol Glycan Core From Trichomonas Vaginalis. Carbohydr. Res. 419, 51–59. 10.1016/j.carres.2015.11.001 26671321 PMC4698206

[B35] Imaizumi T. Yoshida H. Nishi N. Sashinami H. Nakamura T. Hirashima M.. (2007). Double-Stranded RNA Induces Galectin-9 in Vascular Endothelial Cells: Involvement of TLR3, PI3K, and IRF3 Pathway. Glycobiology 17, 12C–15C. 10.1093/glycob/cwm045 17449641

[B36] Jain A. Kumar L. Kushwaha B. Sharma M. Pandey A. Verma V.. (2014). Combining a Synthetic Spermicide With a Natural Trichomonacide for Safe, Prophylactic Contraception. Hum. Reprod. 29, 242–252. 10.1093/humrep/det423 24291662

[B37] King C. C. Jamieson D. J. Wiener J. Cu-Uvin S. Klein R. S. Rompalo A. M.. (2011). Bacterial Vaginosis and the Natural History of Human Papillomavirus. Infect. Dis. Obstet. Gynecol. 2011, 319460. 10.1155/2011/319460 21869857 PMC3159014

[B38] Kissinger P. Amedee A. Clark R. A. Dumestre J. Theall K. P. Myers L.. (2009). Trichomonas Vaginalis Treatment Reduces Vaginal HIV-1 Shedding. Sex Transm. Dis. 36, 11–16. 10.1097/OLQ.0b013e318186decf 19008776 PMC3779369

[B39] Lam K. C. Vyshenska D. Hu J. Rodrigues R. R. Nilsen A. Zielke R. A.. (2018). Transkingdom Network Reveals Bacterial Players Associated With Cervical Cancer Gene Expression Program. PeerJ 6, e5590. 10.7717/peerj.5590 30294508 PMC6170155

[B40] Lanteri M. Giordanengo V. Hiraoka N. Fuzibet J.-G. Auberger P. Fukuda M.. (2003). Altered T Cell Surface Glycosylation in HIV-1 Infection Results in Increased Susceptibility to Galectin-1-Induced Cell Death. Glycobiology 13, 909–918. 10.1093/glycob/cwg110 12925577

[B41] Liang Y. Chen M. Qin L. Wan B. Wang H. (2019). A Meta-Analysis of the Relationship Between Vaginal Microecology, Human Papillomavirus Infection and Cervical Intraepithelial Neoplasia. Infect. Agent Cancer 14, 29. 31673281 10.1186/s13027-019-0243-8 PMC6815368

[B42] Liang K.-Y. Zeger S. (1986). Longitudinal Data Analysis Using Generalized Linear Models. Biometrika 73, 13–22. 10.1093/biomet/73.1.13

[B43] Low A. J. Konate I. Nagot N. Weiss H. A. Kania D. Vickerman P.. (2014). Cervicovaginal HIV-1 Shedding in Women Taking Antiretroviral Therapy in Burkina Faso: A Longitudinal Study. J. Acquir. Immune Defic. Syndr. (1999) 65, 237–245. 10.1097/QAI.0000000000000049 PMC3979829 24226060

[B44] Lustig G. Ryan C. M. Secor W. E. Johnson P. J. (2013). Trichomonas Vaginalis Contact-Dependent Cytolysis of Epithelial Cells. Infect. Immun. 81, 1411–1419. 10.1128/IAI.01244-12 23429535 PMC3648012

[B45] Malla N. Goyal K. Dhanda R. S. Yadav M. (2014). Immunity in Urogenital Protozoa. Parasite Immunol. 36, 400–408. 10.1111/pim.12114 25201404

[B46] Mayer K. H. Hogan J. W. Smith D. Klein R. S. Schuman P. Margolick J. B.. (2003). Clinical and Immunologic Progression in HIV-Infected US Women Before and After the Introduction of Highly Active Antiretroviral Therapy. J. Acquir. Immune Defic. Syndr. (1999) 33, 614–624. 10.1097/00126334-200308150-00011 12902807

[B47] Merani S. Chen W. Elahi S. (2015). The Bitter Side of Sweet: The Role of Galectin-9 in Immunopathogenesis of Viral Infections. Rev. Med. Virol. 25, 175–186. 10.1002/rmv.1832 25760439

[B48] Mercier S. St-Pierre C. Pelletier I. Ouellet M. Tremblay M. J. Sato S. (2008). Galectin-1 Promotes HIV-1 Infectivity in Macrophages Through Stabilization of Viral Adsorption. Virology 371, 121–129. 10.1016/j.virol.2007.09.034 18028978

[B49] Misra J. S. Singh U. (2006). Results of Longterm Hospital Based Cytological Screening in Asymptomatic Women. Diagn. Cytopathol. 34, 184–187. 10.1002/dc.20377 16470854

[B50] Morrison C. Fichorova R. N. Mauck C. Chen P. L. Kwok C. Chipato T.. (2014). Cervical Inflammation and Immunity Associated With Hormonal Contraception, Pregnancy, and HIV-1 Seroconversion. J. Acquir. Immune Defic. Syndr. 66, 109–117. 10.1097/QAI.0000000000000103 24413042

[B51] Norenhag J. Du J. Olovsson M. Verstraelen H. Engstrand L. Brusselaers N. (2020). The Vaginal Microbiota, Human Papillomavirus and Cervical Dysplasia: A Systematic Review and Network Meta-Analysis. Bjog. 127, 171–180. 10.1111/1471-0528.15854 31237400

[B52] Oh H. Y. Kim B. S. Seo S. S. Kong J. S. Lee J. K. Park S. Y.. (2015). The Association of Uterine Cervical Microbiota With an Increased Risk for Cervical Intraepithelial Neoplasia in Korea. Clin. Microbiol. Infect. 21, 674.e671–679. 10.1016/j.cmi.2015.02.026 25752224

[B53] Okumura C. Y. Baum L. G. Johnson P. J. (2008). Galectin-1 on Cervical Epithelial Cells Is a Receptor for the Sexually Transmitted Human Parasite Trichomonas Vaginalis. Cell Microbiol. 10, 2078–2090. 10.1111/j.1462-5822.2008.01190.x 18637021 PMC4437540

[B54] Onderdonk A. B. Delaney M. L. Fichorova R. N. (2016). The Human Microbiome During Bacterial Vaginosis. Clin. Microbiol. Rev. 29, 223–238. 10.1128/CMR.00075-15 26864580 PMC4786887

[B55] Onderdonk A. B. Zamarchi G. R. Rodriguez M. L. Hirsch M. L. Munoz A. Kass E. H. (1987). Qualitative Assessment of Vaginal Microflora During Use of Tampons of Various Compositions. Appl. Environ. Microbiol. 53, 2779–2784. 10.1128/aem.53.12.2779-2784.1987 3435143 PMC204198

[B56] Ouellet M. Mercier S. Pelletier I. Bounou S. Roy J. Hirabayashi J.. (2005). Galectin-1 Acts as a Soluble Host Factor That Promotes HIV-1 Infectivity Through Stabilization of Virus Attachment to Host Cells. J. Immunol. 174, 4120–4126. 10.4049/jimmunol.174.7.4120 15778371

[B57] Provenzano D. Khoshnan A. Alderete J. F. (1997). Involvement of dsRNA Virus in the Protein Composition and Growth Kinetics of Host Trichomonas Vaginalis. Arch. Virol. 142, 939–952. 10.1007/s007050050130 9191859

[B58] Punt S. Thijssen V. L. Vrolijk J. de Kroon C. D. Gorter A. Jordanova E. S. (2015). Galectin-1, -3 and -9 Expression and Clinical Significance in Squamous Cervical Cancer. PLoS One 10, e0129119. 10.1371/journal.pone.0129119 26066796 PMC4467041

[B59] Rabinovich G. A. Toscano M. A. Ilarregui J. M. Rubinstein N. (2004). Shedding Light on the Immunomodulatory Properties of Galectins: Novel Regulators of Innate and Adaptive Immune Responses. Glycoconjugate J. 19, 565–573. 10.1023/B:GLYC.0000014087.41914.72 14758081

[B60] Rodriguez-Cerdeira C. Sanchez-Blanco E. Alba A. (2012). Evaluation of Association Between Vaginal Infections and High-Risk Human Papillomavirus Types in Female Sex Workers in Spain. ISRN Obstet. Gynecol. 2012, 240190. 10.5402/2012/240190 22900198 PMC3415090

[B61] Rowley J. Vander Hoorn S. Korenromp E. Low N. Unemo M. Abu-Raddad L. J.. (2019). Chlamydia, Gonorrhoea, Trichomoniasis and Syphilis: Global Prevalence and Incidence Estimates, 2016. Bullet. World Health Organization 97, 513–519. 10.2471/BLT.18.228486 PMC6653813 31384073

[B62] Sato S. Ouellet M. St-Pierre C. Tremblay M. J. (2012). Glycans, Galectins, and HIV-1 Infection. Ann. N. Y. Acad. Sci. 1253, 133–148. 10.1111/j.1749-6632.2012.06475.x 22524424

[B63] Sato S. St-Pierre C. Bhaumik P. Nieminen J. (2009). Galectins in Innate Immunity: Dual Functions of Host Soluble β-Galactoside-Binding Lectins as Damage-Associated Molecular Patterns (DAMPs) and as Receptors for Pathogen-Associated Molecular Patterns (PAMPs). Immunol. Rev. 230, 172–187. 10.1111/j.1600-065X.2009.00790.x 19594636

[B64] Schroder H. C. Ushijima H. Theis C. Seve A. P. Hubert J. Muller W. E. (1995). Expression of Nuclear Lectin Carbohydrate-Binding Protein 35 in Human Immunodeficiency Virus Type 1-Infected Molt-3 Cells. J. Acquir. Immune Defic. Syndr. Hum. Retrovirol. 9, 340–348. 7600101

[B65] Singh B. N. Hayes G. R. Lucas J. J. Sommer U. Viseux N. Mirgorodskaya E.. (2009). Structural Details and Composition of Trichomonas Vaginalis Lipophosphoglycan in Relevance to the Epithelial Immune Function. Glycoconj. J. 26, 3–17. 10.1007/s10719-008-9157-1 18604640 PMC2637367

[B66] Smith D. K. Warren D. L. Vlahov D. Schuman P. Stein M. D. Greenberg B. L.. (1997). Design and Baseline Participant Characteristics of the Human Immunodeficiency Virus Epidemiology Research (HER) Study: A Prospective Cohort Study of Human Immunodeficiency Virus Infection in US Women. Am. J. Epidemiol. 146, 459–469. 10.1093/oxfordjournals.aje.a009299 9290506

[B67] Stowell S. R. Qian Y. Karmakar S. Koyama N. S. Dias-Baruffi M. Leffler H.. (2008). Differential Roles of Galectin-1 and Galectin-3 in Regulating Leukocyte Viability and Cytokine Secretion. J. Immunol. 180, 3091–3102. 10.4049/jimmunol.180.5.3091 18292532

[B68] Tohill B. C. Heilig C. M. Klein R. S. Rompalo A. Cu-Uvin S. Brown W.. (2004). Vaginal Flora Morphotypic Profiles and Assessment of Bacterial Vaginosis in Women at Risk for HIV Infection. Infect. Dis. Obstet. Gynecol. 12, 121–126. 10.1080/10647440400020711 15763911 PMC1784599

[B69] Trifonova R. T. Doncel G. F. Fichorova R. N. (2009). Polyanionic Microbicides Modify Toll-Like Receptor-Mediated Cervicovaginal Immune Responses. Antimicrob. Agents Chemother. 53, 1490–1500. 10.1128/AAC.01152-08 19139286 PMC2663082

[B70] Van Der Pol B. Kwok C. Pierre-Louis B. Rinaldi A. Salata R. A. Chen P. L.. (2008). Trichomonas Vaginalis Infection and Human Immunodeficiency Virus Acquisition in African Women. J. Infect. Dis. 197, 548–554. 10.1086/526496 18275275

[B71] Viikki M. Pukkala E. Nieminen P. Hakama M. (2000). Gynaecological Infections as Risk Determinants of Subsequent Cervical Neoplasia. Acta Oncol. 39, 71–75. 10.1080/028418600431003 10752657

[B72] Wang S. F. Tsao C. H. Lin Y. T. Hsu D. K. Chiang M. L. Lo C. H.. (2014). Galectin-3 Promotes HIV-1 Budding *via* Association With Alix and Gag P6. Glycobiology 24, 1022–1035. 10.1093/glycob/cwu064 24996823 PMC4181451

[B73] WHO (2012). Global Incidence and Prevalence of Selected Curable Sexually Transmitted Infections - 2008 (Geneva, Switzerland: WHO Press), 20. Available at: http://apps.who.int/iris/bitstream/10665/75181/1/9789241503839_eng.pdf.

[B74] Workowski K. A. (2015). Centers for Disease Control and Prevention Sexually Transmitted Diseases Treatment Guidelines. Clin. Infect. Dis. 61, S759–S762. 10.1093/cid/civ771 26602614

[B75] Yang X. Da M. Zhang W. Qi Q. Zhang C. Han S. (2018). Role of Lactobacillus in Cervical Cancer. Cancer Manag. Res. 10, 1219–1229. 10.2147/CMAR.S165228 29844701 PMC5962305

[B76] Yang M. Li L. Jiang C. Qin X. Zhou M. Mao X.. (2020). Co-Infection With Trichomonas Vaginalis Increases the Risk of Cervical Intraepithelial Neoplasia Grade 2–3 Among HPV16 Positive Female: A Large Population-Based Study. BMC Infect. Dis. 20, 642. 10.1186/s12879-020-05349-0 32873233 PMC7466445

