# Supplementary material for: Protozoan-Viral-Bacterial Co-Infections Alter Galectin Levels and Associated Immunity Mediators in the Female Genital Tract
Source: Front Cell Infect Microbiol. 2021 Aug 5;11:649940. doi: 10.3389/fcimb.2021.649940 (PMC8375472; doi:10.3389/fcimb.2021.649940)
Supplement: Supplementary file 4 [file Table_4.docx]

## Supplemental Table 4 Comparison of case and control visits by non-matched variables

| variable.name | value | Total | TV negative visits | TV positive visits | Test |
| --- | --- | --- | --- | --- | --- |
| PMN | No | 273/446, 61% | 142/223, 64% | 131/223, 59% | Reference |
| PMN | Yes | 173/446, 39% | 81/223, 36% | 92/223, 41% | 1.23 (0.84, 1.80), p=0.29 |
|  |  |  |  |  |  |
| MNC | No | 390/446, 87% | 205/223, 92% | 185/223, 83% | Reference |
| MNC | Yes | 56/446, 13% | 18/223, 8% | 38/223, 17% | 2.33 (1.27, 4.28), p=0.006 |
|  |  |  |  |  |  |
| WBC Group | PMN Only | 124/446, 28% | 66/223, 30% | 58/223, 26% | Reference |
| WBC Group | Both | 49/446, 11% | 15/223, 7% | 34/223, 15% | 2.45 (1.22, 4.89), p=0.011 |
| WBC Group | Neither | 266/446, 60% | 139/223, 62% | 127/223, 57% | 0.99 (0.65, 1.50), p=0.964 |
| WBC Group | MNC only | 7/446, 2% | 3/223, 1% | 4/223, 2% | 1.49 (0.24, 9.13), p=0.665 |
|  |  |  |  |  |  |
| BV (Nugent score) visit prior | no BV (<4) | 157/446, 35% | 77/223, 35% | 80/223, 36% | Reference |
| BV (Nugent score) visit prior | Altered (4-6) | 79/446, 18% | 33/223, 15% | 46/223, 21% | 1.38 (0.74, 2.60), p=0.313 |
| BV (Nugent score) visit prior | BV+ (>6) | 177/446, 40% | 80/223, 36% | 97/223, 43% | 1.33 (0.79, 2.23), p=0.283 |
| BV (Nugent score) visit prior | Missing | 33/446, 7% | 33/223, 15% | 0/223, 0% | - |
|  |  |  |  |  |  |
| HPV at visit | No | 234/446, 52% | 128/223, 57% | 106/223, 48% | Reference |
| HPV at visit | Missing | 1/446, 0% | 0/223, 0% | 1/223, 0% | - |
| HPV at visit | Yes | 211/446, 47% | 95/223, 43% | 116/223, 52% | 1.63 (1.07, 2.48), p=0.023 |
|  |  |  |  |  |  |
| HPV Status (prior/current visit) | -/- | 166/446, 37% | 97/223, 43% | 69/223, 31% | Reference |
| HPV Status (prior/current visit) | Missing/- | 15/446, 3% | 15/223, 7% | 0/223, 0% | - |
| HPV Status (prior/current visit) | Missing/+ | 13/446, 3% | 13/223, 6% | 0/223, 0% | - |
| HPV Status (prior/current visit) | -/+ | 38/446, 9% | 12/223, 5% | 26/223, 12% | 2.74 (1.27, 5.89), p=0.010 |
| HPV Status (prior/current visit) | +/Missing | 1/446, 0% | 0/223, 0% | 1/223, 0% | - |
| HPV Status (prior/current visit) | +/- | 53/446, 12% | 16/223, 7% | 37/223, 17% | 3.35 (1.62, 6.95), p=0.001 |
| HPV Status (prior/current visit) | +/+ | 160/446, 36% | 70/223, 31% | 90/223, 40% | 1.95 (1.16, 3.26), p=0.011 |
|  |  |  |  |  |  |
| HSV-2 status at baseline | Negative | 140/446, 31% | 67/223, 30% | 73/223, 33% | Reference |
| HSV-2 status at baseline | Missing | 10/446, 2% | 4/223, 2% | 6/223, 3% | - |
| HSV-2 status at baseline | Positive | 296/446, 66% | 152/223, 68% | 144/223, 65% | 0.86 (0.56, 1.31), p=0.477 |
|  |  |  |  |  |  |
| Education | <HS | 202/446, 45% | 94/223, 42% | 108/223, 48% | Reference |
| Education | HS | 165/446, 37% | 77/223, 35% | 88/223, 39% | 0.99 (0.65, 1.49), p=0.95 |
| Education | >HS | 79/446, 18% | 52/223, 23% | 27/223, 12% | 0.47 (0.27, 0.80), p=0.006 |
|  |  |  |  |  |  |
| Employed | No | 324/446, 73% | 145/223, 65% | 179/223, 80% | Reference |
| Employed | Yes | 122/446, 27% | 78/223, 35% | 44/223, 20% | 0.41 (0.26, 0.67), p<0.001 |
|  |  |  |  |  |  |
| Medicaid | No | 159/446, 36% | 68/223, 30% | 91/223, 41% | Reference |
| Medicaid | Yes | 287/446, 64% | 155/223, 70% | 132/223, 59% | 0.64 (0.43, 0.95), p=0.026 |
|  |  |  |  |  |  |
| Cigarette smoking | No | 104/446, 23% | 71/223, 32% | 33/223, 15% | Reference |
| Cigarette smoking | Yes | 342/446, 77% | 152/223, 68% | 190/223, 85% | 2.52 (1.58, 4.01), p<0.001 |
|  |  |  |  |  |  |
| Alcohol use | No | 198/446, 44% | 121/223, 54% | 77/223, 35% | Reference |
| Alcohol use | Yes | 248/446, 56% | 102/223, 46% | 146/223, 65% | 2.29 (1.53, 3.44), p<0.001 |
|  |  |  |  |  |  |
| Crack use | No | 357/446, 80% | 196/223, 88% | 161/223, 72% | Reference |
| Crack use | Yes | 88/446, 20% | 27/223, 12% | 61/223, 27% | 2.89 (1.69, 4.94), p<0.001 |
| Crack use | Missing | 1/446, 0% | 0/223, 0% | 1/223, 0% | - |
|  |  |  |  |  |  |
| Cocaine & Heroin use | No | 364/446, 82% | 197/223, 88% | 167/223, 75% | Reference |
| Cocaine & Heroin use | Yes | 81/446, 18% | 26/223, 12% | 55/223, 25% | 2.38 (1.43, 3.97), p=0.001 |
| Cocaine & Heroin use | Missing | 1/446, 0% | 0/223, 0% | 1/223, 0% | - |
|  |  |  |  |  |  |
| Cocaine use alone | No | 337/446, 76% | 187/223, 84% | 150/223, 67% | Reference |
| Cocaine use alone | Yes | 108/446, 24% | 36/223, 16% | 72/223, 32% | 2.64 (1.61, 4.31), p<0.001 |
| Cocaine use alone | Missing | 1/446, 0% | 0/223, 0% | 1/223, 0% | - |
|  |  |  |  |  |  |
| Heroin use alone | No | 329/446, 74% | 186/223, 83% | 143/223, 64% | Reference |
| Heroin use alone | Yes | 116/446, 26% | 37/223, 17% | 79/223, 35% | 2.68 (1.69, 4.25), p<0.001 |
| Heroin use alone | Missing | 1/446, 0% | 0/223, 0% | 1/223, 0% | - |
|  |  |  |  |  |  |
| Any illicit drug | No | 213/446, 48% | 136/223, 61% | 77/223, 35% | Reference |
| Any illicit drug | Yes | 233/446, 52% | 87/223, 39% | 146/223, 65% | 3.11 (2.03, 4.76), p<0.001 |
|  |  |  |  |  |  |
| Vaginal sex with a man | No | 110/446, 25% | 67/223, 30% | 43/223, 19% | Reference |
| Vaginal sex with a man | Yes | 335/446, 75% | 156/223, 70% | 179/223, 80% | 1.96 (1.20, 3.21), p=0.008 |
| Vaginal sex with a man | Missing | 1/446, 0% | 0/223, 0% | 1/223, 0% | - |
|  |  |  |  |  |  |
| Male partners | None | 110/445, 25% | 67/223, 30% | 43/222, 19% | Reference |
| Male partners | One | 240/445, 54% | 113/223, 51% | 127/222, 57% | 1.92 (1.15, 3.22), p=0.013 |
| Male partners | Two | 59/445, 13% | 28/223, 13% | 31/222, 14% | 1.90 (0.98, 3.67), p=0.057 |
| Male partners | 3+ | 36/445, 8% | 15/223, 7% | 21/222, 9% | 2.38 (1.09, 5.22), p=0.03 |
|  |  |  |  |  |  |
| Condom use with a male | All the time | 126/446, 28% | 53/223, 24% | 73/223, 33% | Reference |
| Condom use with a male | Missing | 4/446, 1% | 2/223, 1% | 2/223, 1% | - |
| Condom use with a male | No sex | 110/446, 25% | 67/223, 30% | 43/223, 19% | 0.41 (0.23, 0.75), p=0.004 |
| Condom use with a male | Not all the time | 206/446, 46% | 101/223, 45% | 105/223, 47% | 0.74 (0.47, 1.17), p=0.201 |
|  |  |  |  |  |  |
| CD4+ T-cell count | <200 | 57/296, 19% | 27/148, 18% | 30/148, 20% | Reference |
| CD4+ T-cell count | >=200 & <350 | 153/296, 52% | 75/148, 51% | 78/148, 53% | 0.93 (0.48, 1.79), p=0.828 |
| CD4+ T-cell count | >=350 | 80/296, 27% | 44/148, 30% | 36/148, 24% | 0.72 (0.36, 1.47), p=0.372 |
| CD4+ T-cell count | Missing | 6/296, 2% | 2/148, 1% | 4/148, 3% | 1.88 (0.31, 11.44), p=0.492 |
|  |  |  |  |  |  |
| HIV plasma viral load | <=500 | 72/296, 24% | 42/148, 28% | 30/148, 20% | Reference |
| HIV plasma viral load | >500 & <=10,000 | 138/296, 47% | 65/148, 44% | 73/148, 49% | 1.54 (0.86, 2.76), p=0.149 |
| HIV plasma viral load | >10,000 | 83/296, 28% | 41/148, 28% | 42/148, 28% | 1.39 (0.72, 2.66), p=0.328 |
| HIV plasma viral load | Missing | 3/296, 1% | 0/148, 0% | 3/148, 2% | - |
|  |  |  |  |  |  |
| ARV | HAART | 43/296, 15% | 28/148, 19% | 15/148, 10% | Reference |
| ARV | Missing | 47/296, 16% | 11/148, 7% | 36/148, 24% | - |
| ARV | No ARV | 112/296, 38% | 62/148, 42% | 50/148, 34% | 1.64 (0.80, 3.35), p=0.175 |
| ARV | Non-HAART | 94/296, 32% | 47/148, 32% | 47/148, 32% | 1.87 (0.88, 3.96), p=0.104 |
|  |  |  |  |  |  |
|  |  |  |  |  |  |
